# Providing Virtual Psychosocial and Trauma Support to Displaced Women in Nigeria: A Case Study of Courage, Innovation and Collaboration; Core Values and Behaviours of the Royal College of Psychiatrists

**DOI:** 10.1192/bjo.2025.10753

**Published:** 2025-06-20

**Authors:** Udoka Onyechere, Toibat Adeyinka, Eghonghon Abumere, Ada Ugochukwu

**Affiliations:** 1 Cumbria, Northumberland, Tyne and Wear Foundation NHS Trust, Newcastle, United Kingdom; 2 Coventry and Warwickshire Partnership NHS Trust, Coventry, United Kingdom; 3 Leicester Partnership NHS Trust, Leicester, United Kingdom

## Abstract

**Aims:** Internally displaced persons (IDPs) in Nigeria experience numerous challenges, including psychological distress resulting from displacement and trauma. In October 2024, a cohort of volunteer mental health professionals based in the United Kingdom provided sessions of mental health support via virtual means to displaced women in Abuja, Nigeria. This intervention utilised telemedicine to address barriers to access and adhered to the principles of Psychological First Aid (PFA).

**Methods:** The initiative required significant pre-event coordination. Mental health professionals from local and international backgrounds were recruited to volunteer, and a structured counselling protocol was developed. Zoom conferencing was utilised to conduct the sessions, with efforts made to incorporate trauma-informed approaches. Despite thorough planning, technical difficulties arose, including unreliable Wi-Fi connections that intermittently disrupted sessions. Power outages were also problematic, necessitating the use of generators at the Nigerian location to maintain a supportive environment. During the sessions, participants disclosed accounts of hardship and trauma. Volunteers emphasised active listening, empathy, and cultural sensitivity to establish trust and provide meaningful support.

**Results:** Collaboration played a pivotal role in the success of this initiative, showcasing its critical contribution to the field of mental health care. The collaboration between local and international mental health professionals underscored the strength of teamwork. Each volunteer’s dedication, despite the challenges, contributed to the overall impact of the project. The shared responsibility among the team fostered a supportive network that bolstered resilience against challenges, which is key in mental health-care provision. Feedback from participants and volunteers reflected the success of the initiative, with attendees expressing gratitude and an openness to future mental health interventions.

**Conclusion:** This initiative demonstrated the power of perseverance, creativity, and collaborative efforts in delivering psychosocial support to underserved populations. By adopting a flexible approach and prioritising the needs of the participants, the event successfully bridged gaps in care and provided a platform for displaced women to be heard and supported. Future initiatives can build upon these findings, enhancing technological resilience and refining to ensure greater reach to those most in need.

